# Novel Carboranyl Derivatives of Nucleoside Mono- and Diphosphites and Phosphonates: A Synthetic Investigation

**DOI:** 10.1155/S1565363304000032

**Published:** 2004

**Authors:** Kamesh Vyakaranam, Narayan S. Hosmane

**Affiliations:** Department of Chemistry and Biochemistry Michael Faraday Laboratories Northern Illinois University DeKalb Illinois 60115-2862 USA

## Abstract

A number of nucleoside mono- and diphosphites and phosphonates containing 1,2-dicarbadodecaborane (12) (la-6b) at 5'-position of the sugar moiety have been synthesized in good yields. Experimental details along with the spectroscopic and analytical data, supporting the formation of the title compounds, are presented. These constitute a new generation of boron compounds that are envisioned to be useful in cancer treatment *via* Boron Neutron Capture Therapy (BNCT).


### Novel Carboranyl Derivatives of Nucleoside Mono- and Diphosphites and Phosphonates: A Synthetic Investigation

Kamesh Vyakaranam and Narayan S. Hosmane*

*Department of Chemistry and Biochemistry, Michael Faraday Laboratories, Northern Illinois University, DeKalb, Illinois 60115-2862*
**E-mail: nhosmane@niu.edu*

### ABSTRACT

A number of nucleoside mono- and diphosphites and phosphonates containing 1,2-dicarbadodecaborane (12) (**1a-6b**) at 5'-position of the sugar moiety have been synthesized in good yields. Experimental details along with the spectroscopic and analytical data, supporting the formation of the title compounds, are presented. These constitute a new generation of boron compounds that are envisioned to be useful in cancer treatment *via* Boron Neutron Capture Therapy (BNCT).

### INTRODUCTION

The concept of BNCT was first proposed by Locher some sixty years ago /1/. However, the successful application of BNCT for the treatment of cancer still presents a challenge in medical research. Over the last decade or so, various boron-containing analogues of biologically active compounds such as amino acids /2/, peptides /3/, porphyrins /4/, polyamines /5/, nucleosides, as well as DNA binders /6/, have been synthesized and evaluated for their possible use in BNCT. Such analogues might function in a manner similar to their naturally occurring counterparts and become selectively incorporated into either proliferating or more metabolically active tumor cells. The requirement of 25-30$\mu g$ of $^{10}B$ atoms per gram of tumor for effective BNCT /7/ has led researchers to incorporate 1, 2-dicarbadodecaborane (*o*-carborane) as the integral part of many cancer drugs /8/. The $C_2B_{10}$ carborane cage has unique electronic and steric properties that also make it an intriguing moiety in drug design /9/. Boron containing nucleosides are potentially attractive because they should be (1) taken up selectively into tumor due to high mitotic rate of tumor cells vs. normal cells, (2) *intra*-cellularly converted to the corresponding nucleotides by phosphorylation, and (3) incorporated into tumor DNA, thereby enhancing the cytotoxicity of neutron capture therapy /10/. Early work focused on the development and synthesis of boron-containing purine and pyrimidine bases in which the boron atom was placed within the purine or pyrimidine nucleus and flanked by two nitrogen atoms /11/. Several boron containing nucleosides have also been synthesized with a borane, cyanoborane, dihydroxylboryl, and carboranyl unit attached to a nucleic acid base /12/. Nonetheless, an ideal BNCT drug should exhibit the hydrolytic stability of the $P\text{-}C_{(cage)}$-nucleotide linkages under physiological conditions without substantial cytotoxicity to the normal cells. Therefore, construction of such linkages is warranted. Consequently, our preliminary results indicated that several prototype carboranyl-substituted adenosine diphosphate (ADP) can be synthesized and their chemistry explored /13/. Thus, this work led us to explore similar species containing carborane cages at 5'-position of the sugar moiety with the hope of serving a dual purpose in providing a significant boron concentration as well as mimicing the naturally occurring nucleosides in the living system /2,3/12/. Here we report the syntheses and characterization of novel carboranyl derivatives of nucleoside mono- and diphosphites and phosphonates.

### EXPERIMENTAL

### Materials

All solvents, chemicals and reagents were of analytical grade and used without further purification unless otherwise noted. Baker analyzed silica gel (60-200 mesh) was used for flash column chromatography. 1,2-Bis(chlorophenylphosphino)-1,2-dicarbadodecaborane (**1**), 1-phenyl-2-(chlorophenylphosphino)-1,2-dicarba-dodecaborane (**2**), 1-methyl-2-(chlorophenylphosphino)-1,2-dicarbadodecaborane (**3**) were prepared by the methods described elsewhere /14/. 1,2-Bis(chloromethylphosphate)-1,2-dicarbadodecaborane (**4**), 1-phenyl-2-(chloromethylphosphate)-1,2-dicarbadodecaborane (**5**), 1-methyl-2-(chloromethylphosphate)-1,2-dicarba-dodecaborane (**6**) were synthesized by the methods described in the literature /15/. *Closo*-1,2-$C_2B_{10}H_{12}$ (*o*-carborane), *closo*-1-Ph-1,2-$C_2B_{10}H_{11}$ (phenyl-*o*-carborane), and *closo*-1-Me-1,2-$C_2B_{10}H_{11}$ (methyl-*o*-carborane) were obtained from KATCHEM and used as received. $N^6$-3'-O-dibenzoyl-2'-deoxyadenosine, $N^2$-isobutyryl-3'-acetyl-2'-deoxyguanosine, phenyl dichlorophosphine and methyl dichlorophosphate were obtained from Sigma-Aldrich and used without further purification. Tetrahydrofuran (THF), triethylamine (TEA) were dried over sodium metal and benzophenone and doubly distilled before use.

### Spectroscopic and analytical procedures

The $^1H$, $^{11}B$, $^{31}P$ and $^{13}C$ NMR spectra were recorded on a Bruker Fourier-transform multinuclear NMR spectrometer at 200, 64.2, 80.2 and 50.3 MHz, respectively. Infrared spectra were recorded using a Nicolet Magna 550 FT-IR spectrophotometer with OMNIC software. Elemental analyses were obtained in house using a Perkin Elmer 2400 CHN elemental analyzer.

### Synthetic procedures

All experiments were carried out in 100 mL Pyrex glass round-bottom flasks of each fitted with a nitrogen inlet and containing a magnetic stirring bar. All known compounds among the products were identified by comparing their IR and NMR spectra and melting points with those of authentic samples.

**1,2-Bis[phenylphosphino-$N^6$-3'-O-dibenzoyl-2'-deoxyadenosine]-1,2-dicarbadodeca-borane (1a)**

A 0.28 mmol (0.12 g) sample of 1,2-bis(chlorophenylphosphino)-1,2-dicarbadodecaborane (**1**), $N^6$-3'-O-dibenzoyl-2'-deoxyadenosine (0.56 mmol, 0.26 g), and triethylamine (0.58 mmol, 0.057 g) was dissolved in anhydrous THF in an inert atmosphere and the mixture was stirred for 10 h. After removing the insoluble materials by filtration, the solvent in the filtrate was removed under reduced pressure and the resulting residue was dissolved in chloroform (30 mL) and washed with water (3 x 20 mL) in an extraction procedure. The organic layer in the extract was dried over $MgSO_4$, filtered and concentrated under reduced pressure to produce a solid residue that was purified by column chromatography, using silica gel and a solvent mixture of ethyl acetate: hexane (7:3) as an eluent, to isolate **1a** as a yellow solid in 52 % yield (0.146 mmol, 0.19 g). M. P. >200 °C, dec. Spectroscopic and Analytical Data: $^1H$ NMR (DMSO, relative to $Me_4Si$) δ 8.31 [s, 2H, H-2], 8.01 [s, 2H, H-8], 7.60-8.65 [m, 30H, aromatic H], 6.32 [s, 1H, H-1'], 4.79 [m, 2H, H-3'], 4.22 [m, 2H, H-4'], 3.95 [m, 4H, H-5'], 2.39 [m, 4H, H-2'], 1.0-1.4 [br, 10H, BH]; $^{11}B$ NMR (DMSO, relative to $BF_3.OEt_2$) δ -14.63 [2B, $J_{(BH)}$= 173.0 Hz], -13.12 [4B, $J_{(BH)}$=159.5 Hz], -9.53 [2B, $J_{(BH)}$=153.3 Hz], -2.34 [2B, $J_{(BH)}$=138.0 Hz]; $^{31}P$ NMR (DMSO, relative to $H_3PO_4$) δ 7.60 [s]; $^{13}C$ NMR (DMSO, relative to $Me_4Si$) δ 129.00-143.30 [aromatic], 178.00 [C=O], 70.70 [carborane C]; IR (KBr pellet, $cm^{-1}$) 2576 [ν(B-H)], 705, 1621, 1476 [ν(aromatic)]. Elemental Anal. Calcd. for $C_{62}H_{60}P_2B_{10}N_{10}O_{10}$: C, 58.37; H, 4.75; N, 10.98. Found: C, 58.45; H, 4.66; N, 11.21.

**1,2-Bis[phenylphosphino-$N^2$-isobutyryl-3'-acetyl-2'-deoxyguanosine]-1,2 dicarbadodeca-borane (1b)**

In an inert atmosphere, a 0.28 mmol (0.12 g) sample of 1,2-bis(chlorophenylphosphino)-1,2-dicarbadodecaborane (**1**), $N^2$-isobutyryl-3'-acetyl-2'-deoxyguanosine (0.57 mmol, 0.21 g), and triethylamine (0.59 mmol, 0.057 g) were dissolved in anhydrous THF and the mixture was stirred for 18 h. After removing the insoluble materials by filtration, the solvent in the filtrate was removed under reduced pressure and the resulting residue was extracted with chloroform (25 mL) and washed with water (3 x 15 mL). The organic layer in the extract was dried over $MgSO_4$, filtered and concentrated under reduced pressure to produce a solid residue that was purified by column chromatography, using silica gel and a solvent mixture of ethyl acetate: hexane: pentane (7:2:1) as an eluent, to isolate **1b** as an off-white solid in 58 % yield (0.162 mmol, 0.17 g). M. P. >200 °C dec. Spectroscopic and Analytical Data: $^1H$ NMR (DMSO, relative to $Me_4Si$) δ 8.12 [s, 2H, H-8], 7.60-8.15 [br, 10H, aromatic], 6.41 [m, 2H, H-1'], 4.82 [m, 2H, H-3']; 4.12 [m, 2H, H-4'], 3.92 [4H, m, H-5'], 3.20 [dd, 4H, $CH_2$], 2.41 [m, 4H, H-2'], 2.30 [s, 6H, $CH_3$], 1.50 [m, 2H, CH], 1.20 [br, 10H, BH], 1.10 [d, 6H, $CH_3$], 0.90 [d, 6H, $CH_3$]; $^{11}B$ NMR (DMSO, relative to $BF_3.OEt_2$) δ -14.51 [2B, $J_{(BH)}$= 176.0 Hz], -13.43 [4B, $J_{(BH)}$=162.0 Hz], -9.62 [2B, $J_{(BH)}$=151.0 Hz], -2.58 [2B, $J_{(BH)}$=141.0 Hz]; $^{31}P$ NMR (DMSO, relative to $H_3PO_4$) δ 7.20 [s]; $^{13}C$ NMR (DMSO, relative to $Me_4Si$) δ 172.00 [C=O], 128.70 [aromatic], 72.51 [carborane C]; IR (KBr pellet, $cm^{-1}$) 2583 [ν(B-H)], 711, 1627, 1479 [ν(aromatic)]. Elemental Anal. Calcd. for $C_{46}H_{64}P_2B_{10}N_{10}O_{10}$: C, 50.80; H, 5.94; N, 12.88. Found: C, 50.89; H, 6.10; N, 12.75.

**1-Phenyl-2-[phenylphosphino-$N^6$-3'-O-dibenzoyl-2'-deoxyadenosine]-1,2-dicarbadodecaborane (2a)**

In a procedure identical to that described above for **1a** and **1b**, the reaction involving 0.28 mmol (0.10 g) of 1-phenyl-2-(chlorophenylphosphino)-1,2-dicarbadodecaborane (**2**), $N^6$-3'-O-dibenzoyl-2'-deoxyadenosine (0.30 mmol, 0.13 g), and triethylamine (0.32 mmol, 0.028 g) in anhydrous THF produced **2a** as a white solid in 62 % yield (0.171 mmol, 0.13 g). M. P. >173 °C dec. Spectroscopic and Analytical Data: $^1$H NMR (DMSO, relative to $Me_4Si$) δ 8.08 [s, 1H, H-8], 7.99 [s, 1H, H-2], 7.48-8.50 [m, 20H, aromatic H], 6.28 [m, 1H, H-1'], 4.68 [m, 1H, H-3'], 4.18 [m, 1H, H-4'], 3.96 [m, 2H, H-5'], 2.31 [m, 2H, H-2'], 1.10-1.30 [br, 10H, BH]; $^{11}$B NMR (DMSO, relative to $BF_3.OEt_2$) δ -14.36 [4B, $J_{(BH)}$ = unresolved], -11.89 [2B, $J_{(BH)}$ = 156.8 Hz], -9.56 [2B, $J_{(BH)}$ =151.4 Hz], -7.42 [1B, $J_{(BH)}$ =135.8 Hz], -5.21 [1B, $J_{(BH)}$ =143.4 Hz]; $^{31}$P NMR (DMSO, relative to $H_3PO_4$) δ 8.10 [s]; $^{13}$C NMR (DMSO, relative to $Me_4Si$) δ 177.80 [C=O], 130.00-141.00 [aromatic], 82.00 [carborane C]; IR (KBr pellet, $cm^{-1}$) 2489 [ν(B-H)], 710, 1611, 1471 [ν(aromatic)]. Elemental Anal. Calcd. for $C_{38}H_{40}PB_{10}N_5O_5$: C, 58.06; H, 5.13; N, 8.91. Found: C, 58.21; H, 4.99; N, 8.88.

**1-Phenyl-2-[phenylphosphino-$N^2$-isobutyryl-3'-acetyl-2'-deoxyguanosine]-1,2-dicarbadodecaborane (2b)**

In a procedure identical to that described above for **1a** and **1b**, the reaction of 0.28 mmol (0.10 g) of 1-phenyl-2-(chlorophenylphosphino)-1,2-dicarbadodecaborane (**2**), $N^2$-isobutyryl-3'-acetyl-2'-deoxyguanosine (0.29 mmol, 0.10 g), and triethylamine (0.31 mmol, 0.028 g) in anhydrous THF produced **2b** as a white solid in 59 % yield (0.162 mmol, 0.11 g). M. P. >190 °C dec. Spectroscopic and Analytical Data: $^1$H NMR (DMSO, relative to $Me_4Si$) δ 8.12 [s, 1H, H-8], 7.86 [m, 10H, Ar-H], 6.11 [m, 1H, H-1'], 4.60 [m, 1H, H-3'], 4.21 [m, 1H, H-4'], 3.98 [m, 2H, H-5'], 3.21 [m, 2H, $CH_2$], 2.36 [s, 3H, $CH_3$], 2.33 [m, 2H, H-2'], 1.32 [m, 1H, CH], 1.20-1.41 [br, 10H, B-H], 0.90, 1.30 [d, 6H, $CH_3$]; $^{11}$B NMR (DMSO, relative to $BF_3.OEt_2$) δ -14.32 [4B, $J_{(BH)}$ = unresolved], -12.02 [2B, $J_{(BH)}$ = 158.0 Hz], -10.12 [2B, $J_{(BH)}$ =153.8 Hz], -7.56 [1B, $J_{(BH)}$ =138.6 Hz], -5.09 [1B, $J_{(BH)}$ =141.2 Hz]; $^{31}$P NMR (DMSO, relative to $H_3PO_4$) δ 8.22 [s]; $^{13}$C NMR (DMSO, relative to $Me_4Si$) δ 168.00 [C=O], 129.41 [aromatic], 81.68, 71.60 [carborane C]; IR (KBr pellet, $cm^{-1}$) 2495 [ν(B-H)], 710, 1625, 1482 [ν(aromatic)]. Elemental Anal. Calcd. for $C_{30}H_{42}PB_{10}N_5O_5$: C, 52.06; H, 6.12; N, 10.12. Found: C, 52.10; H, 6.14; N, 10.22.

**1-Methyl-2-[phenylphosphino-$N^6$-3'-O-dibenzoyl-2'-deoxyadenosine]-1,2-dicarbadodecaborane (3a)**

In a procedure identical to that described above, the reaction of 0.43 mmol (0.13 g) of 1-methyl-2-(chlorophenylphosphino)-1,2-dicarbadodecaborane (**3**) with $N^6$-3'-O-dibenzoyl-2'-deoxyadenosine (0.45 mmol, 0.20 g), and triethylamine (0.48 mmol, 0.044 g) in anhydrous THF produced **3a** as a pale yellow solid in 57 % yield (0.25 mmol, 0.18 g). M. P. >200 °C, dec. Spectroscopic and Analytical Data: $^1$H NMR (DMSO, relative to $Me_4Si$) δ 8.12 [s, 1H, H-2], 7.95 [s, 1H, H-8], 7.45-8.55 [m, 15H, Ar-H], 6.31 [m, 1H, H-1'], 4.68 [m, 1H, H-3'], 4.25 [m, 1H, H-4'], 3.86 [m, 2H, H-5'], 2.42 [m, 2H, H-2'], 2.10 [s, 3H, $CH_3$], 1.10-1.35 [br,

10H, B-H]; [11]B NMR (DMSO, relative to $BF_3.OEt_2$) δ -15.92 [4B, $J_{(BH)}$ = unresolved], -13.47 [2B, $J_{(BH)}$ = 161.6 Hz], -10.95 [2B, $J_{(BH)}$=149.00 Hz], -8.75 [1B, $J_{(BH)}$=146.2 Hz], -6.33 [1B, $J_{(BH)}$=153.8 Hz],; [31]P NMR (DMSO, relative to $H_3PO_4$) δ 7.95 [s]; [13]C NMR (DMSO, relative to $Me_4Si$) δ 176.00 [C=O], 131.00-143.50 [aromatic], 82.61, 74.62 [carborane C]; IR (KBr pellet, $cm^{-1}$) 2570 [ν(B-H)], 711, 1634, 1468 [ν(aromatic)]. Elemental Anal. Calcd. for $C_{33}H_{38}PB_{10}N_5O_5$: C, 54.74; H, 5.29; N, 9.67. Found: C, 54.70; H, 5.33; N, 9.55.

### 1-Methyl-2-[phenylphosphino-N$^2$-isobutyryl-3'-acetyl-2'-deoxyguanosine]-1,2-dicarbadodecaborane (3b)

In a procedure identical to that described above, the reaction involving 0.43 mmol (0.13 g) of 1-methyl-2-(chlorophenylphosphino)-1,2-dicarbadodecaborane (**3**) with N$^2$-isobutyryl-3'-acetyl-2'-deoxyguanosine (0.49 mmol, 0.17 g), and triethylamine (0.50 mmol, 0.044 g) in anhydrous THF produced **3b** as a pink solid in 56 % yield (0.241 mmol, 0.15 g). M. P. >200 °C, dec. Spectroscopic and Analytical Data: [1]H NMR (DMSO, relative to $Me_4Si$) δ 8.61 [s, 1H, H-8], 7.86 [m, 5H, Ar-H], 5.98 [m, 1H, H-1'], 4.81 [m, 1H, H-3'], 4.16 [m, 1H, H-4'], 3.87 [m, 2H, H-5'], 3.25 [dd, 2H, $CH_2$], 2.40 [m, 2H, H-2'], 2.10, 2.65 [s, 6H, $CH_3$], 2.10 [m, 1H, CH], 1.20-1.40 [br, 10H, B-H], 1.10, 1.80 [d, 6H, $CH_3$]; [11]B NMR (DMSO, relative to $BF_3.OEt_2$) δ -16.20 [4B, $J_{(BH)}$ = unresolved], -13.76 [2B, $J_{(BH)}$ = 163.8 Hz], -10.76 [2B, $J_{(BH)}$=148.6 Hz], -8.58 [1B, $J_{(BH)}$=143.7 Hz], -6.15 [1B, $J_{(BH)}$=151.5 Hz]; [31]P NMR (DMSO, relative to $H_3PO_4$) δ 8.12 [s]; [13]C NMR (DMSO, relative to $Me_4Si$) δ 172.56 [C=O], 131.50 [aromatic], 80.65, 73.72 [carborane C]; IR (KBr pellet, $cm^{-1}$) 2574 [ν(B-H)], 712, 1628, 1473 [ν(aromatic)]. Elemental Anal. Calcd. for $C_{25}H_{40}PB_{10}N_5O_5$: C, 47.66; H, 6.41; N, 11.12. Found: C, 47.77; H, 6.30; N, 11.33.

### 1,2-Bis[methylphosphate-N$^6$-3'-O-dibenzoyl-2'-deoxyadenosine]-1,2-dicarbadodecaborane (4a)

In a procedure identical to that described above, the reaction of 0.35 mmol (0.13 g) of 1,2-bis (chloromethylphosphate)-1,2-dicarbadodecaborane (**4**), N$^6$-3'-O-dibenzoyl-2'-deoxyadenosine (0.73 mmol, 0.32 g), and triethylamine (0.75 mmol, 0.072 g) in anhydrous THF produced a solid residue that was extracted with dichloromethane (15 mL) and washed with water (3 x 20 mL). The separation and the purification steps, as described above for **1a** and **1b**, produced **4a** as an off-white solid in 58 % yield (0.202 mmol, 0.25 g). M. P. >195 °C, dec. Spectroscopic and Analytical Data: [1]H NMR (DMSO, relative to $Me_4Si$) δ 8.38 [m, 20H, Ar-H], 8.32 [s, 2H, H-8], 5.79 [m, 2H, H-1'], 4.79 [m, 2H, H-4'], 4.48 [m, 2H, H-3'], 3.90 [s, 6H, $OCH_3$], 3.81 [m, 4H, H-5'], 2.39 [m, 4H, H-2'], 1.12-1.31 [br, 10H, BH]; [11]B NMR (DMSO, relative to $BF_3.OEt_2$) δ -14.21 [2B, $J_{(BH)}$ = 174.6 Hz], -13.38 [4B, $J_{(BH)}$=158.6 Hz], -10.07 [2B, $J_{(BH)}$=142.8 Hz], -2.61 [2B, $J_{(BH)}$=129.5 Hz]; [31]P NMR (DMSO, relative to $H_3PO_4$) δ 8.47 [s]; [13]C NMR (DMSO, relative to $Me_4Si$) δ 174.30 [C=O], 145.60 [aromatic], 81.65 [carborane C], 58.00 [$OCH_3$]; IR (KBr pellet, $cm^{-1}$) 2588 [ν(B-H)], 716, 1628, 1481 [ν(aromatic)]. Elemental Anal. Calcd. for $C_{52}H_{56}P_2B_{10}N_{10}O_{14}$: C, 51.38; H, 4.64; N, 11.52. Found: C, 51.77; H, 4.63; N, 11.11.

**1,2-Bis[methylphosphate-N$^2$-isobutyryl-3'-acetyl-2'-deoxyguanosine]-1,2-dicarbadodecaborane (4b)**

In a procedure identical to that described above, the reaction of 0.35 mmol (0.13 g) of 1,2-bis (chloromethylphosphate)-1,2-dicarbadodecaborane (4), N$^2$-isobutyryl-3'-acetyl-2'-deoxyguanosine (0.72 mmol, 0.27 g), and triethylamine (0.74 mmol, 0.072 g) in anhydrous THF produced a solid residue that was extracted with a 1:1 mixture of dichloromethane:chloroform (15 mL) and washed with water (3 x 15 mL). The separation and the purification steps, as described above for **1a** and **1b**, produced **4b** as an off-white solid in 56 % yield (0.197 mmol, 0.20 g). M. P. >220 °C, dec. Spectroscopic and Analytical Data: $^1$H NMR (DMSO, relative to $Me_4Si$) δ 8.62 [s, 2H, H-8], 6.51 [m, 2H, H-1'], 4.86 [m, 2H, H-4'], 4.51 [m, 2H, H-3'], 3.98 [s, 6H, $OCH_3$], 3.76 [m, 4H, H-5'], 3.21 [m, 4H, $CH_2$], 2.42 [m, 4H, H-2'], 2.12 [s, 6H, $CH_3$], 1.82 [m, 2H, CH], 1.00-1.32 [br, 10H, BH], 0.9, 1.3 [d, 12H, $CH_3$]; $^{11}$B NMR (DMSO, relative to $BF_3.OEt_2$) δ -14.32 [2B, $J_{(BH)}$ = 173.0 Hz], -13.21 [4B, $J_{(BH)}$=159.6 Hz], -10.21 [2B, $J_{(BH)}$=144.0 Hz], -2.68 [2B, $J_{(BH)}$=132.0 Hz]; $^{31}$P NMR (DMSO, relative to $H_3PO_4$) δ 8.50 [s]; $^{13}$C NMR (DMSO, relative to $Me_4Si$) δ 176.00 [C=O], 78.65 [carborane C], 59.80 [$OCH_3$]; IR (KBr pellet, cm$^{-1}$) 2570 [ν(B-H)]. Elemental Anal. Calcd. for $C_{36}H_{60}P_2B_{10}N_{10}O_{14}$: C, 42.08; H, 5.89; N, 13.63. Found: C, 42.18; H, 6.01; N, 13.72.

**1-Phenyl-2-[methylphosphate-N$^6$-3'-O-dibenzoyl-2'-deoxyadenosine]-1,2-dicarbadodecaborane (5a)**

In a procedure identical to that described above, the reaction of 0.36 mmol (0.12 g) of 1-phenyl-2-(chloromethylphosphate)-1,2-dicarbadodecaborane (5), N$^6$-3'-O-dibenzoyl-2'-deoxyadenosine (0.38 mmol, 0.16 g), and triethylamine (0.39 mmol, 0.038 g) in anhydrous THF produced a solid residue that was extracted with ethyl acetate (25 mL) and washed with water (3 x 20 mL). The separation and the purification steps, as described above for **1a** and **1b**, produced **5a** as a yellowish-gray solid in 54 % yield (0.194 mmol, 0.15 g). M. P. >175 °C, dec. Spectroscopic and Analytical Data: $^1$H NMR (DMSO, relative to $Me_4Si$) δ 8.16 [s, 1H, H-8], 7.95 [s, 1H, H-2], 7.40-8.43 [m, 15H, Ar-H], 6.36 [m, 1H, H-1'], 4.82 [m, 1H, H-4'], 4.51 [m, 1H, H-3'], 3.86 [s, 3H, $OCH_3$], 3.83 [m, 2H, H-5'], 2.31 [m, 2H, H-2'], 1.00-1.30 [br, 10H, B-H]; $^{11}$B NMR (DMSO, relative to $BF_3.OEt_2$) δ -14.17 [4B, unresolved], -11.82 [2B, $J_{(BH)}$ = 153.7 Hz], -9.16 [2B, $J_{(BH)}$ =146.7 Hz], -7.13 [1B, $J_{(BH)}$=136.4 Hz], -4.98 [1B, $J_{(BH)}$=141.6 Hz]; $^{31}$P NMR (DMSO, relative to $H_3PO_4$) δ 8.37 [s]; $^{13}$C NMR (DMSO, relative to $Me_4Si$) δ 174.85 [C=O], 128.50-142.50 [aromatic], 81.75, 73.80 [carborane C], 59.67 [$OCH_3$]; IR (KBr pellet, cm$^{-1}$) 2565 [ν(B-H)], 710, 1622, 1478 [ν(aromatic)]. Elemental Anal. Calcd. for $C_{33}H_{38}PB_{10}N_5O_7$: C, 52.42; H, 5.07; N, 9.26. Found: C, 52.58; H, 4.97; N, 9.28.

**1-Phenyl-2-[methylphosphate-N$^2$-isobutyryl-3'-acetyl-2'-deoxyguanosine]-1,2-dicarbadodecaborane (5b)**

In a procedure identical to that described above, the reaction of 0.36 mmol (0.12 g) of 1-phenyl-2-(chloromethylphosphate)-1,2-dicarbadodecaborane (5), N$^2$-isobutyryl-3'-acetyl-2'-deoxyguanosine (0.39 mmol, 0.14 g), and triethylamine (0.42 mmol, 0.038 g) in anhydrous THF produced a solid residue that was extracted with a 1:2 mixture of ethyl acetate:chloroform (25 mL) and washed with water (3 x 15 mL). The separation and the purification steps, as described above for **1a** and **1b**, produced **5b** as a yellow solid in 58 % yield (0.208 mmol, 0.14 g). M. P. >200 °C, dec. Spectroscopic and Analytical Data: $^{1}$H NMR (DMSO, relative to $Me_4Si$) δ 8.64 [s, 1H, H-8], 7.43 [m, 5H, Ar-H], 6.48 [m, 1H, H-1'], 4.83 [m, 1H, H-4'], 4.48 [m, 1H, H-3'], 3.99 [s, 3H, $OCH_3$], 3.75 [m, 2H, H-5'], 2.45 [m, 2H, H-2'], 2.43 [m, 2H, $CH_2$], 2.30 [s, 3H, $CH_3$], 1.86 [m, 1H, CH], 0.90, 1.40 [d, 6H, $CH_3$], 1.00-1.30 [br, 10H, B-H]; $^{11}$B NMR (DMSO, relative to $BF_3.OEt_2$) δ -14.36 [4B, unresolved], -11.68 [2B, $J_{(BH)}$= 157.3 Hz], -9.35 [2B, $J_{(BH)}$=143.8 Hz], -7.42 [1B, $J_{(BH)}$=135.8 Hz], -4.87 [1B, $J_{(BH)}$=143.5 Hz]; $^{31}$P NMR (DMSO, relative to $H_3PO_4$) δ 8.39 [s]; $^{13}$C NMR (DMSO, relative to $Me_4Si$) δ 171.50 [C=O], 129.60 [aromatic], 78.87, 82.60 [carborane C], 60.15 [$OCH_3$]; IR (KBr pellet, $cm^{-1}$) 2568 [ν(B-H)], 713, 1626, 1473 [ν(aromatic)]. Elemental Anal. Calcd. for $C_{25}H_{40}PB_{10}N_5O_7$: C, 45.35; H, 6.09; N, 10.58. Found: C, 45.34; H, 6.12; N, 10.55.

### 1-Methyl-2-[methylphosphate-N$^{6}$-3'-O-dibenzoyl-2'-deoxyadenosine]-1,2-dicarbadodecaborane (6a)

In a procedure identical to that described above, the reaction of 0.29 mmol (0.08 g) of 1-methyl-2-(chloromethylphosphate)-1,2-dicarbadodecaborane (**6**), N$^{6}$-3'-O-dibenzoyl-2'-deoxyadenosine (0.33 mmol, 0.13 g), and triethylamine (0.36 mmol, 0.030 g) in anhydrous THF produced a solid residue that was extracted with chloroform (25 mL) and washed with water (3 x 15 mL). The separation and the purification steps, as described above for **1a** and **1b**, produced **6a** as a pale yellow solid in 52 % yield (0.150 mmol, 0.11 g). M. P. >175 °C, dec. Spectroscopic and Analytical Data: $^{1}$H NMR (DMSO, relative to $Me_4Si$) δ 8.48 [m, 10H, Ar-H], 1.10-1.30 [br, 10H, B-H], 2.15 [s, 3H, $CH_3$], 3.90 [s, 3H, $OCH_3$], 8.21 [s, 1H, H-2], 8.01 [s, 1H, H-8], 6.42 [m, 1H, H-1'], 2.38 [m, 2H, H-2'], 4.55 [m, 1H, H-3'], 4.76 [m, 1H, H-4'], 3.88 [m, 2H, H-5']; $^{11}$B NMR (DMSO, relative to $BF_3.OEt_2$) δ -5.92 [1B, $J_{(BH)}$=156.4 Hz], -7.83 [1B, $J_{(BH)}$=148.7 Hz], -11.12 [2B, $J_{(BH)}$=154.8 Hz], -14.13 [2B, $J_{(BH)}$= 172.4 Hz], -16.83 [4B, unresolved]; $^{31}$P NMR (DMSO, relative to $H_3PO_4$) δ 8.42 [s]; $^{13}$C NMR (DMSO, relative to $Me_4Si$) δ 146.75 [aromatic], 83.80, 73.44 [carborane C], 57.00 [$OCH_3$], 175.00 [C=O]; IR (KBr pellet, $cm^{-1}$) 2561 [ν(B-H)], 715, 1627, 1474 [ν(aromatic)]. Elemental Anal. Calcd. for $C_{28}H_{36}PB_{10}N_5O_7$: C, 48.46; H, 5.23; N, 10.09. Found: C, 48.50; H, 5.39; N, 9.98.

### 1-Methyl-2-[methylphosphate-N$^{2}$-isobutyryl-3'-acetyl-2'-deoxyguanosine]-1,2-dicarbadodecaborane (6b)

In a procedure identical to that described above, the reaction of 0.29 mmol (0.08 g) sample of 1-methyl-2-(chloromethylphosphate)-1,2-dicarbadodecaborane (**6**), N$^{2}$-isobutyryl-3'-acetyl-2'-deoxyguanosine (0.31 mmol, 0.11 g), and triethylamine (0.35 mmol, 0.030 g) in anhydrous THF produced a solid residue that was extracted with 1:2 mixture of ethyl acetate:chloroform (20 mL) and washed with water (3 x 20 mL). The separation and the purification steps, as described above for **1a** and **1b**, produced **6b** as a yellow solid in 54 % yield (0.157 mmol, 0.09 g). M. P. >220 °C, dec. Spectroscopic and Analytical Data: $^{1}$H NMR (DMSO, relative to $Me_4Si$) δ 8.28 [s, 1H, H-8], 5.86 [m, 1H, H-1'], 4.75 [m, 1H, H-3'], 4.08 [m, 1H, H-4'], 3.95 [s,

3H, $OCH_3$], 3.88 [m, 2H, H-5'], 3.23 [m, 2H, $CH_2$], 2.42 [m, 2H, H-2'], 2.20, 1.60 [s, 6H, $CH_3$], 1.80 [dd, 1H, CH], 1.00-1.30 [br, 10H, B-H], 0.90, 1.30 [d, 6H, $CH_3$]; $^{11}B$ NMR (DMSO, relative to $BF_3.OEt_2$) δ -16.69 [4B, unresolved], -14.03 [2B, $J_{(BH)}$= 171.3 Hz], -10.89 [2B, $J_{(BH)}$=153.6 Hz], -7.76 [1B, $J_{(BH)}$=147.9 Hz], -5.87 [1B, $J_{(BH)}$=157.2 Hz]; $^{31}P$ NMR (DMSO, relative to $H_3PO_4$) δ 8.46 [s]; $^{13}C$ NMR (DMSO, relative to $Me_4Si$) δ 178.00 [C=O], 78.68, 71.65 [carborane C], 59.00 [$OCH_3$]; IR (KBr pellet, $cm^{-1}$) 2560 [ν(B-H)]. Elemental Anal. Calcd. for $C_{20}H_{38}PB_{10}N_5O_7$: C, 40.04; H, 6.39; N, 11.67. Found: C, 39.99; H, 6.22; N, 11.53.

### RESULTS AND DISCUSSION

We recently reported the synthesis of carboranyl bis(adenosine diphosphate) (CBADP) in which diphosphorylated adenosine base was attached to both the carbons of the carborane cage. The specific synthetic route involved the reaction of 1,2-bis(methyl dichlorophosphate)-1,2-dicarbadodecaborane with 5'-methoxy monophosphorylated-3'-O-and $N^6$-protected deoxyadenosine /13/. However, the present work involves the syntheses of a number of carboranyl nucleosides in which the phosphino or phosphate substituted bases are attached either to one carbon or both the carbons of the carborane cage, instead of the diphosphorylated bases. In addition, the present work has been performed with two bases (adenosine and guanosine). The synthetic route has been simplified by one step reaction involving the chlorophenylphosphino-substituted and chloromethylphosphate-substituted carboranes with adenosine or guanosine to yield the desired species, **1a-6b** as shown in **Schemes 1-2**.

1,2-Bis[phenylphosphinodeoxynucleoside]-1,2-dicarbadodecaborane, **1a-b**, and 1-phenyl or methyl-2-[phenylphosphinodeoxynucleoside]-1,2-dicarbadodecaborane, **2a-b** or **3a-b**, were obtained in moderate yields (56-62 %) from the reaction of 3'-O- and $N^6$-protected deoxyadenosine, or from similarly protected deoxyguanosine, with the disubstituted (phenylchlorophosphine)carborane or the monosubstituted (phenylchlorophosphine)methyl- or phenyl carborane with triethylamine in THF. Protection of the base nitrogens in deoxyadenosine and deoxyguanosine was also important to prevent boronation at the base positions. All of the carboranyl derivatives of mono- and diphosphites and phosphonates have been characterized by their elemental analyses, NMR spectra and IR spectra. The $^{31}P$ NMR resonance was observed between 7.00-8.25 ppm as a broad singlet, presumably due to a long-range coupling of the phosphorus with the boron atoms of the cage. It is of interest to note that the $^{31}P$ chemical shifts for a number of *closo-o*-carboranylphosphines appear in the range of 5.4 ppm to 54.2 ppm depending on the substituent on the phosphorus atom /16/. On the other hand, the $^{11}B$ NMR chemical shifts of *closo-o*-carboranylphosphines appear in the range of −1 ppm to −13 ppm /16/, and are almost similar to those of **1a** – **3b** whose $^{11}B$ NMR chemical shifts appeared in the range of −2.00 to −16.00 ppm indicating the presence of similar cage functionality on the cage carbons in these compounds. The IR spectra of **1a** – **3b** exhibited the characteristic B-H stretching absorptions in the region of 2560-2583 $cm^{-1}$ that indicates the presence of carborane cages in these species. The $^{13}C$ NMR spectra, in addition to the resonances due to alkyl and/or aromatic functionalities in the expected region of chemical shifts, showed peaks in the region of δ 70.0 - 86.0 ppm due to cage carbons of the *closo-o*-carborane unit and are quite close to the values of δ = 65.1 – 84.5 ppm observed for

**Scheme 1:** Synthesis of 1,2-bis[phenylphosphinodeoxynucleoside]-1,2-dicarbadodecaborane and 1-phenyl or methyl-2-[phenylphosphinodeoxynucleoside]-1,2-dicarbadodecaborane.

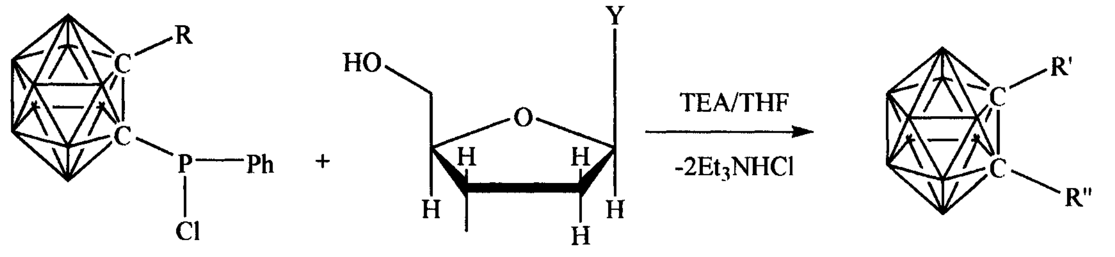

1. R= P(Ph)Cl
2. R= Ph
3. R= Me

R'' =

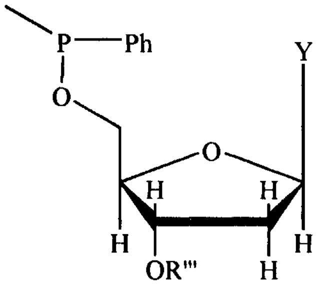

1a. R' = R''; R''' = Bz; Y = Ade
1b. R' = R''; R''' = Ac; Y = Gua
2a. R' = Ph; R''' = Bz; Y = Ade
2b. R' = Ph; R''' = Ac; Y = Gua
3a. R' = Me; R''' = Bz; Y = Ade
3b R' = Me; R''' = Ac; Y = Gua

Ade =

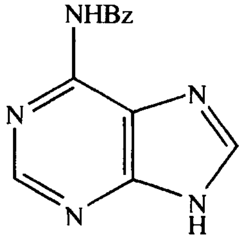

Gua =

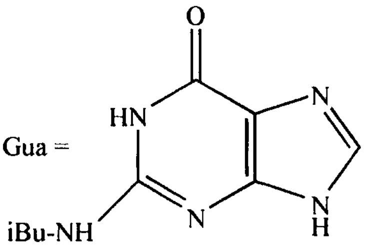

*closo-o*-carboranylphosphines /16/. The proton NMR spectra were consistent with all of the moieties present in **1a**–**3b**.

The compounds 1,2-bis[methylphosphatedeoxynucleoside]-1,2-dicarbadodeca-borane, **4a-b**, and 1-phenyl or methyl-2-[methylphosphatedeoxy-nucleoside]-1,2-dicarbadodecaborane, **5a-b** or **6a-b**, were obtained in 50-58 % yield from the reaction of the appropriate deoxynucleoside and the disubstituted (chloromethylphosphate), or the mono-substituted (chloromethylphosphate) carborane. The deprotection of the protecting groups on the bases in the final compounds was not tried. All of the products shown in **Scheme 2** have been characterized by elemental analyses, $^{1}$H, $^{11}$B, $^{13}$C and $^{31}$P NMR spectra and IR spectra (see Experimental section). The chemical shift values of $\delta$ = 8.25 ppm in the $^{31}$P NMR spectra, $\delta$ = –2.61 to –16.38 ppm in the $^{11}$B NMR spectra, and $\delta$ = 71.0 - 83.6 ppm in the $^{13}$C NMR spectra, in addition to those in the $^{1}$H NMR spectra, are all in the range observed for the recently reported carboranyl bis(adenosine diphosphate) /13/ indicating the presence of similar linkages that exist between the *exo*-polyhedral moieties and the *closo-o*-carboranyl cage in **4a-b**, **5a-b** and **6a-b**. The IR spectra showed the characteristic B-H stretching frequencies in the region of 2565-2580 cm$^{-1}$. Nonetheless, the utility of these compounds as a tumor-targeting species for effective boron neutron capture therapy (BNCT) or in photodynamic therapy (PDT) will be investigated in the near future. Since all of the reaction sequences, described in **Schemes 1-2**, are of a general nature, they represent a blueprint for the synthesis of a family of potentially valuable hydrolytically stable carborane nucleic acid constructs. Studies evaluating the *in-vitro* and *in-vivo* localizing ability of these novel compounds are currently being carried out in our laboratories.

### ACKNOWLEDGEMENT

This work was supported by grants from the National Science Foundation (CHE-9988045, CHE-0241319), the donors of the Petroleum Research Fund, administered by the American Chemical Society, and Northern Illinois University through a Presidential Research Profesorship. The Forschungspreis der Alexander von Humboldt-Stiftung is also hereby gratefully acknowledged.

### REFERENCES

1. G.L. Locker, *Am. J. Roentgenol. Radiat. Ther.*, **36**, 1 (1936).
2. a. P.A. Radel and S.B. Kahl, *J. Org. Chem.*, **61**, 4582 (1996) and references therein; b. I.H. Hall, S. Hall, L.K. Chi, B.R. Shaw, A. Sood and B.F. Spielvogel, *Anticancer Res.*, **12**, 1091 (1992).
3. a. R.R. Kane and M.F. Hawthorne, *Bioconjugate Chem.*, **2**, 241 (1991); b. A. Sood, B.F. Spielvogel and B.R. Shaw, *J. Am. Chem. Soc.*, **111**, 9234 (1989).
4. G. Oenbrink, P. Jurgenlimke and D. Gabel, *Photochem. Photobiol.*, **48**, 451 (1988).
5. a. H. Ghaneolhosseini, W. Tjarks and S. Sjoberg, *Tetrahedron*, **54**, 3877 (1998); b. H. Ghaneolhosseini, W. Tjarks and S. Sjoberg, *Tetrahedron*, 17519 (1997).

**Scheme 2:** Synthesis of 1,2-bis[methylphosphatedeoxynucleoside]-1,2-dicarbadodecaborane and 1-phenyl or methyl-2-[methylphosphatedeoxynucleoside]-1,2-dicarbadodecaborane.

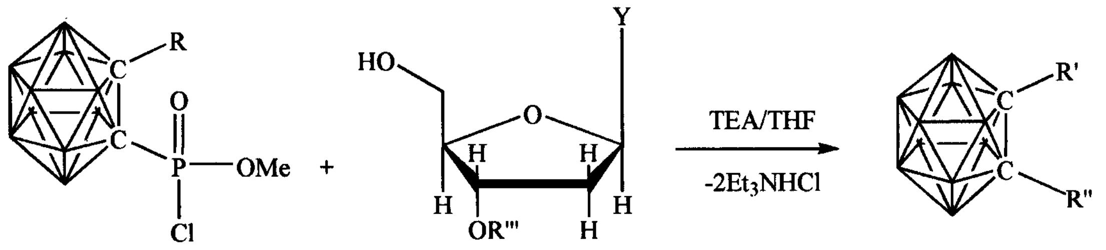

4. R= P(O)(OMe)Cl

5. R= Ph

6. R= Me

R'' =

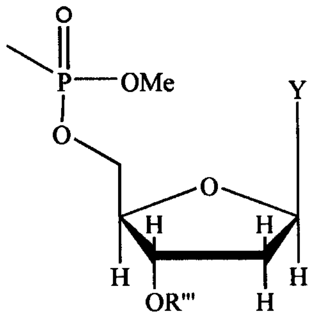

4a. R' = R''; R''' = Bz; Y = Ade
4b. R' = R''; R''' = Ac; Y = Gua
5a. R' = Ph; R''' = Bz; Y = Ade
5b. R' = Ph; R''' = Ac; Y = Gua
6a. R' = Me; R''' = Bz; Y = Ade
6b R' = Me; R''' = Ac; Y = Gua

Ade =

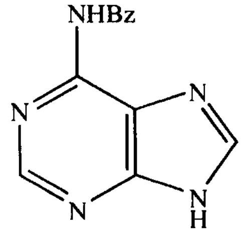

Gua =

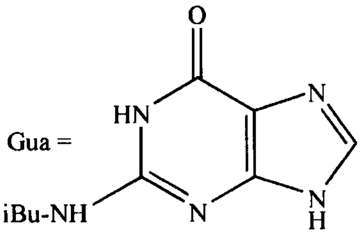

6. a. T. Hartman and J. Carlsson, *Radiother. Oncol.*, **31**, 61 (1994); b. D. Gabel, S. Foster and R.G. Fairchild, *Radiat. Res.*, **111**, 14 (1987).
7. M.F. Hawthorne, *Angew. Chem. Int. Ed. Engl.*, **32**, 950 (1993).
8. a. R.N. Grimes, *Carboranes*, Academic Press, New York, 1970; b. V.I. Bregardze, *Chem. Rev.*, **92**, 209 (1992); c. G. Hondrogiannis and G.W. Kabalka, *Tetrahedron Lett.*, **36**, 4365 (1995).
9. Y. Endo, *Contemporary Boron Chemistry*, The Royal Society of Chemistry, Cambridge, U.K., 2000; p. 127.
10. Y. Yamamoto, T. Seko, H. Nakamura, H. Nemoto, H. Hojo and N. Hashimoto, *J. Chem. Soc., Chem. Commun.*, **1992**, 157.
11. a. M.P. Groziak, A.D. Ganguly and P.D. Robinson, *J. Am. Chem. Soc.*, **116**, 7597 (1994); b. D.S. Matteson and T.C. Cheng, *J. Org. Chem.*, **33**, 3055 (1968); c. A. Maitra, *Indian J. Chem.*, **16B**, 85 (1978).
12. a. T.K. Liao, E.G. Pondrebarac and C.C. Cheng, *J. Am. Chem. Soc.*, **86**, 1869 (1964); b. R.F. Schinazi and W.H. Prusoff, *Tetrahedron Lett.*, **50**, 4891 (1978); c. Z.J. Lesnikowski and R.F. Schinazi, *J. Org. Chem.*, **58**, 6531 (1993); d. Z.J. Lesnikowski, R.M. Lloyd Jr. and R.F. Schinazi, *Nucleosides Nucleotides*, **16**, 1503 (1997); e. G. Fulcrand-El Kattan, Z.J. Lesnikowski, S. Yao, F. Tanious, W.D. Wilson and R.F. Schinazi, *J. Am. Chem. Soc.*, **116**, 7494 (1994); f. Z.J. Lesnikowski, G. Fulcrand, R.M. Lloyd, Jr., A. Juodawlkis and R.F. Schinazi, *Biochemistry*, **35**, 5741 (1996).
13. K. Vyakaranam, G. Rana, S. Delaney, S. Ledger and N.S. Hosmane, *Inorg. Chem. Comm.*, **6**, 654 (2003).
14. R.P. Alexander and H.A. Schroeder, *Inorg. Chem.*, **2**, 1107 (1963).
15. R.A. Bechtold and A. Kaczmarczyk, *J. Med. Chem.*, **18**, 371 (1975).
16. R. Nunez, C. Vinas, F. Teixidor, R. Sillanpâä and R. Kivekâs, *J. Organomet. Chem.*, **592**, 22 (1999).

